# Synchronous Nodular Epithelial Hyperplasia and Type A Microthymoma Adjacent to a Thymic Cyst: A Report of a Rare Case

**DOI:** 10.7759/cureus.116724

**Published:** 2026-09-22

**Authors:** Yujiro Bunno, Marino Yamamoto, Ryohei Miyazaki, Hironobu Okada, Masaya Tamura

**Affiliations:** 1 Department of Thoracic Surgery, Kochi Medical School, Kochi University, Nankoku, JPN; 2 Department of Thoracic Surgery, Kochi Medical School Hospital, Nankoku, JPN

**Keywords:** case report, microscopic thymoma, thymic cyst, thymic epithelial proliferation, type a microthymoma

## Abstract

Thymic cysts are uncommon benign lesions of the anterior mediastinum and are frequently detected incidentally on imaging studies. Microscopic thymoma and microthymoma are extremely rare microscopic thymic epithelial proliferations that are usually identified only by histopathological examination. Their relationship to thymic cysts and to each other remains poorly understood.

A 54-year-old woman was referred to our institution after an anterior mediastinal lesion was incidentally detected on routine chest computed tomography (CT). Magnetic resonance imaging and fluorodeoxyglucose positron emission tomography/CT findings were consistent with a thymic cyst. Robot-assisted thoracoscopic resection of the cyst with the adjacent thymic tissue was performed for definitive diagnosis and treatment. Histopathological examination revealed a unilocular thymic cyst accompanied by two incidental microscopic thymic epithelial lesions within the adjacent thymic tissue: a 0.6-mm microscopic thymoma and a 2.0-mm Type A microthymoma. Based on their characteristic size and histological features, both lesions were diagnosed without additional immunohistochemical analysis. The postoperative course was uneventful, and the patient remains free of recurrence 18 months after surgery.

To our knowledge, the coexistence of microscopic thymoma and Type A microthymoma adjacent to a thymic cyst is exceptionally rare. The close proximity and similar histological features of these lesions suggest that they may represent different stages within a continuous morphological spectrum of microscopic thymic epithelial proliferations rather than entirely distinct entities. Furthermore, this case highlights the potential value of careful pathological examination of thymic tissue adjacent to resected thymic cysts for detecting occult microscopic epithelial lesions and improving pathological assessment.

## Introduction

Thymic cysts are uncommon benign lesions of the anterior mediastinum and are often detected incidentally during imaging examinations performed for unrelated reasons. In contrast, microscopic thymic epithelial proliferations are usually identified incidentally on histopathological examination. Lesions historically termed “microscopic thymoma” are now generally regarded as nodular epithelial hyperplasia (NEH), a benign microscopic proliferation of thymic epithelial cells rather than a subtype of conventional thymoma. Microthymoma, in contrast, refers to a small thymic epithelial neoplasm that shows architectural and cytological features resembling those of conventional thymoma. Type A microthymoma is characterized by a compact proliferation of bland spindle-shaped or oval thymic epithelial cells with morphological features resembling those of conventional Type A thymoma. Distinguishing NEH from microthymoma is important because of their overlapping histopathological features and their different biological classifications. Although awareness of these entities has increased in recent years, their pathogenesis, biological significance, and relationship to each other, as well as their association with thymic cysts, remain poorly understood.

Although the coexistence of thymic cysts with microscopic thymic epithelial proliferations has been reported, cases demonstrating both NEH and Type A microthymoma in close proximity within the same surgical specimen remain uncommon. The present case represents another rare example and provides an opportunity to further consider the possible biological relationship between these lesions.

Herein, we report a rare case in which NEH (historically termed microscopic thymoma) and Type A microthymoma were incidentally identified adjacent to a thymic cyst following surgical resection. We describe the clinicopathological findings and discuss the possible relationship between these microscopic thymic epithelial lesions and thymic cysts in the context of previously reported cases.

## Case presentation

A 54-year-old woman with a medical history of dyslipidemia and non-alcoholic fatty liver disease was referred to our institution after an anterior mediastinal lesion was incidentally detected during a routine health-screening chest computed tomography (CT) examination. She was asymptomatic and had no clinical findings suggestive of myasthenia gravis.

Contrast-enhanced chest CT demonstrated a well-defined, approximately 4.0 × 3.0 cm nodular lesion with an irregular contour in the anterior mediastinum (Figure 1A). Magnetic resonance imaging (MRI) showed low signal intensity on T1-weighted imaging and homogeneous high signal intensity on T2-weighted imaging, findings consistent with a cystic lesion containing fluid (Figure 1B). ^18^F-fluorodeoxyglucose positron emission tomography/computed tomography (FDG PET/CT) demonstrated no abnormal FDG uptake within the lesion. No focal mural thickening, enhancing solid component, or surrounding solid nodule was identified. Based on these imaging findings, including the cystic characteristics and absence of metabolic activity, a thymic cyst was strongly suspected. The lesion measured 4.0 × 3.0 cm, with no focal mural thickening or surrounding solid nodules.

**Figure 1:**
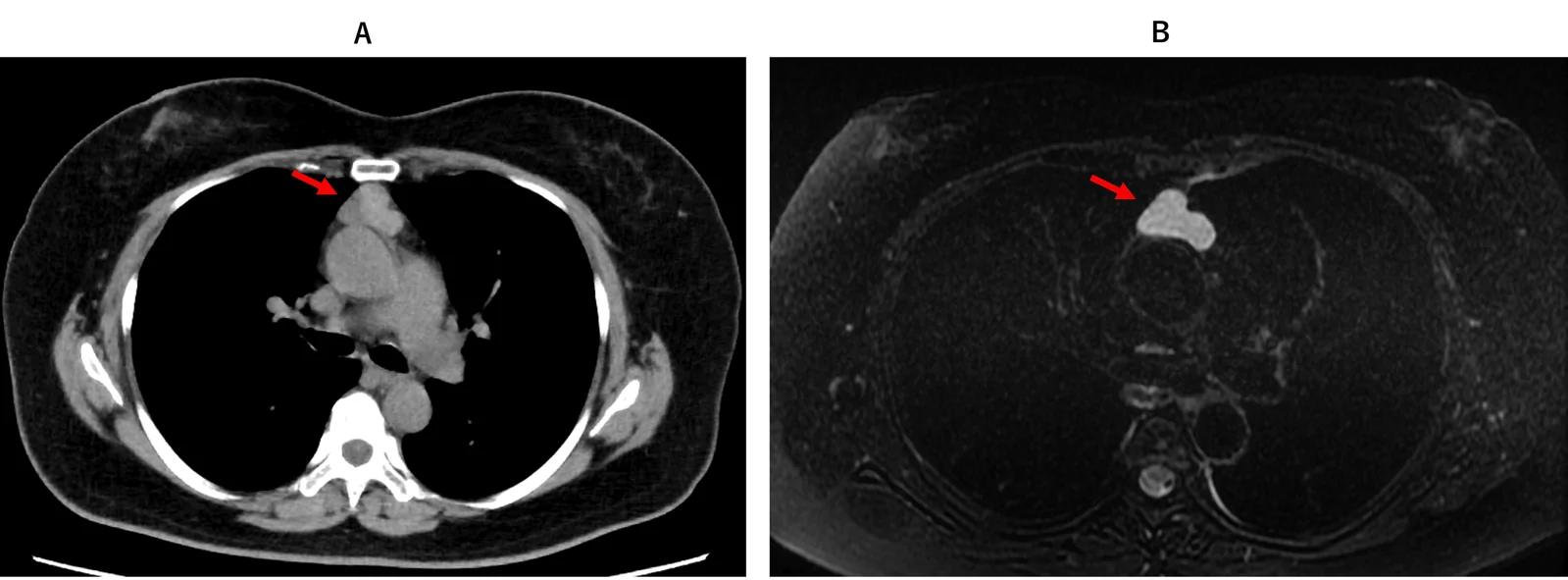
Preoperative imaging findings (A) Contrast-enhanced chest computed tomography demonstrated a well-defined cystic lesion measuring 4.0 × 3.0 cm in the anterior mediastinum (red arrow). No focal mural thickening or associated solid nodule was identified. (B) Magnetic resonance imaging showed homogeneous high signal intensity on T2-weighted imaging (red arrow), findings consistent with a cystic lesion.

Laboratory investigations were unremarkable except for mildly elevated serum aspartate aminotransferase (AST) and alanine aminotransferase (ALT) levels. The serum AST and ALT levels were 47 U/L and 68 U/L, respectively, while the anti-acetylcholine receptor antibody (anti-AChR antibody) level was less than 0.2 nmol/L (Table 1).

**Table 1:** Laboratory findings at presentation AST, aspartate aminotransferase; ALT, alanine aminotransferase; anti-AChR antibody, anti-acetylcholine receptor antibody.

Laboratory parameter	Result	Reference range	Unit
Aspartate aminotransferase (AST)	47	13-30	U/L
Alanine aminotransferase (ALT)	68	7–23	U/L
Anti-acetylcholine receptor antibody (anti-AChR antibody)	<0.2	<0.4	nmol/L

The patient had no symptoms or physical findings suggestive of myasthenia gravis, including muscle weakness. Based on these findings, a thymic cyst was considered the most likely preoperative diagnosis. Although the imaging findings were suggestive of a benign thymic cyst, definitive exclusion of an epithelial neoplasm could not be achieved by imaging alone. In addition, the lesion was relatively large and located in the anterior mediastinum. Therefore, surgical resection was performed for definitive pathological diagnosis and treatment. Robot-assisted thoracoscopic resection of the cyst together with the immediately adjacent thymic tissue was performed. The lesion was completely separated from the surrounding mediastinal structures, and no gross invasion or involvement of adjacent organs was observed. A complete thymectomy was not performed because the lesion was localized and the preoperative diagnosis was a thymic cyst.

The resected specimen was oriented and submitted for pathological examination, including the cyst wall and the adjacent residual thymic tissue. The specimen was examined in two tissue blocks, and a total of six histological levels/sections were evaluated. Both lesions were located within the residual thymic tissue immediately adjacent to the cyst wall. Careful examination of the residual thymic tissue identified two minute epithelial proliferative foci in close proximity to the cyst wall (Figure 2).

**Figure 2:**
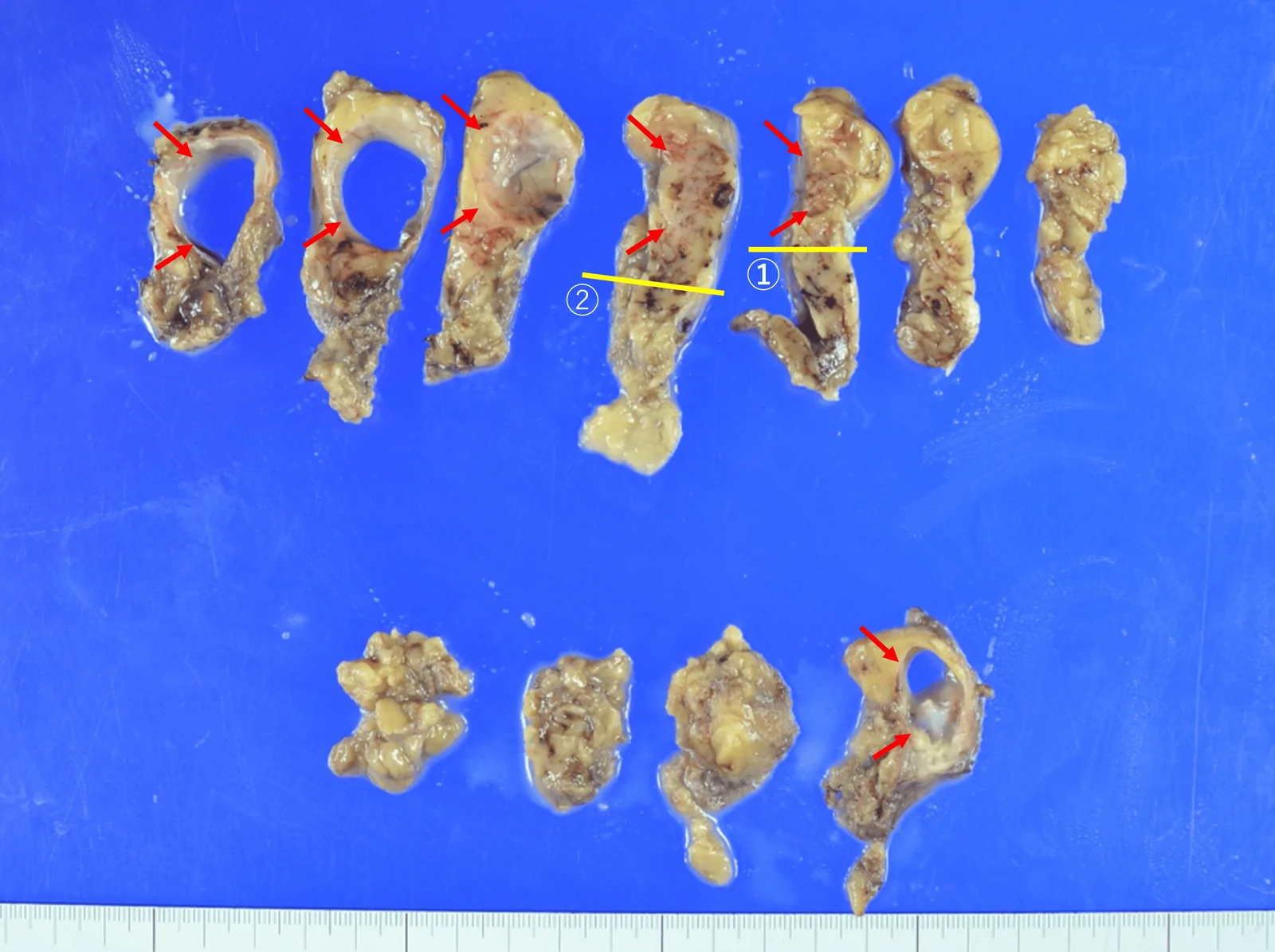
Macroscopic findings of the resected specimen Serial sections of the resected specimen demonstrated a unilocular thymic cyst (red arrow). Two microscopic thymic epithelial lesions were identified within the adjacent thymic tissue. Lesion ① corresponds to the Type A microthymoma found in the histopathology results, whereas lesion ② corresponds to the microscopic thymoma.

Histopathological examination demonstrated a 4.0 × 3.0 cm unilocular cyst embedded within predominantly adipose tissue. The cyst was lined by ciliated columnar epithelium with an underlying fibrous connective tissue layer. Importantly, no cartilage, bronchial-type glands, or prominent smooth muscle components were identified in the cyst wall. Residual thymic tissue containing Hassall corpuscles was identified adjacent to the cyst wall, supporting the diagnosis of a thymic cyst rather than a bronchogenic cyst.

Within the residual thymic tissue, two well-circumscribed microscopic epithelial proliferations were identified. The smaller lesion measured 0.6 × 0.4 mm and consisted of a localized proliferation of bland thymic epithelial cells without significant cytological atypia, destructive growth, or features diagnostic of conventional thymoma. In accordance with current terminology, this lesion was classified as nodular epithelial hyperplasia (NEH), historically termed “microscopic thymoma.” The lesion was located approximately 1.4 mm from the cyst wall. The second lesion measured 2.0 × 0.6 mm and showed a discrete, compact proliferation of bland spindle-shaped to oval thymic epithelial cells with morphological features resembling those of conventional Type A thymoma (Figure 3). The lesion showed no prominent lobulation, no definite capsule, and no definite perivascular spaces. No significant cytological atypia, mitotic activity, necrosis, or destructive stromal invasion was identified. The lesion was completely surrounded by thymic tissue, with no involvement of the examined surgical margin. The lesion was located approximately 0.8 mm from the cyst wall. Taken together, the compact thymoma-like architecture and the characteristic bland spindle-shaped to oval epithelial cells supported the diagnosis of Type A microthymoma rather than NEH.

**Figure 3:**
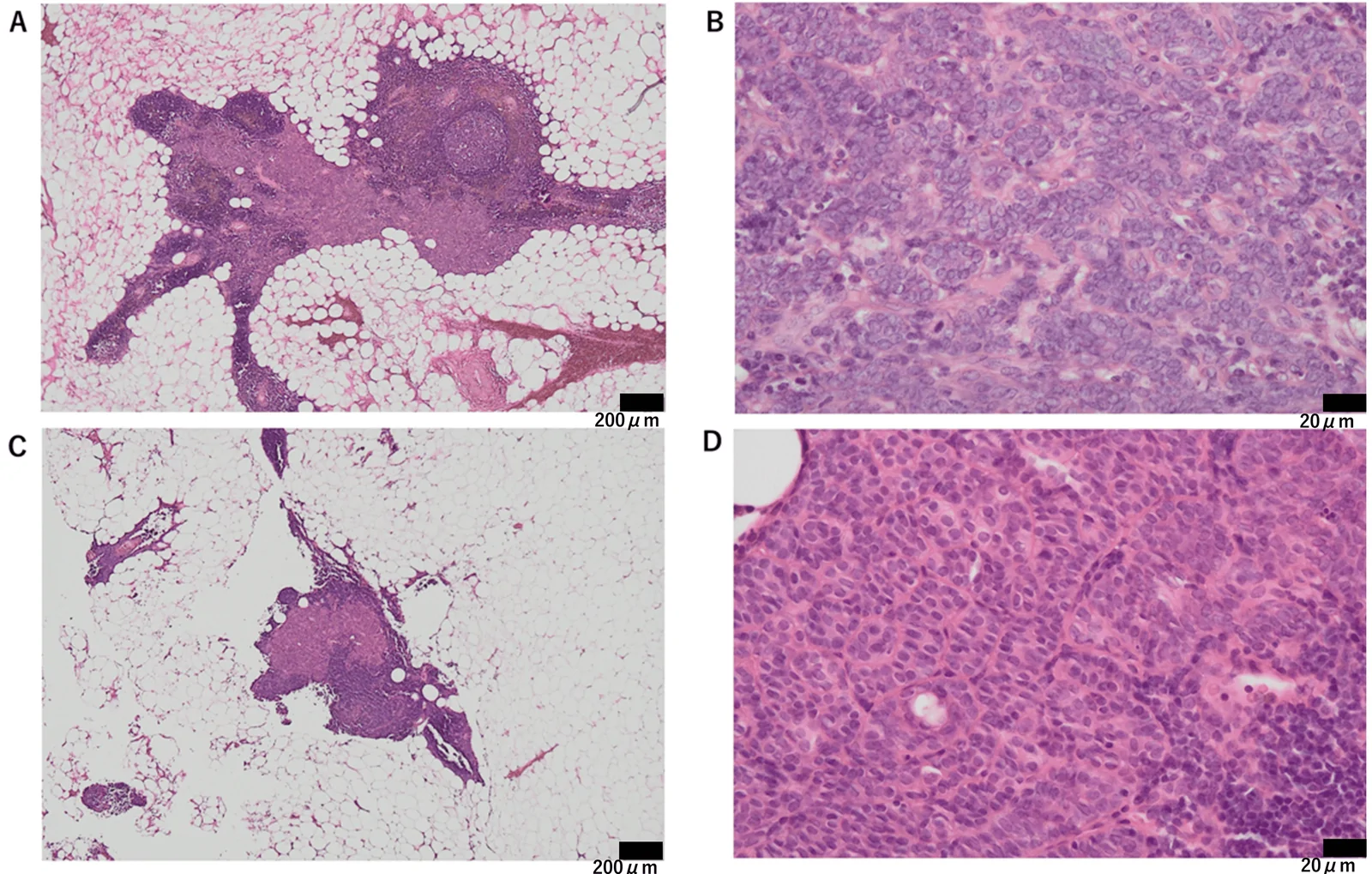
Histopathological findings on H&E staining (A) Low-power photomicrograph of lesion ① in Figure 2 showing a well-circumscribed 2.0 × 0.6 mm proliferation of thymic epithelial cells. (B) High-power view of lesion ① demonstrating a solid proliferation of cytologically bland spindle-shaped thymic epithelial cells, consistent with Type A microthymoma. (C) Low-power photomicrograph of lesion ② in Figure 2 showing a minute 0.6 × 0.4 mm focus of thymic epithelial cell proliferation. (D) High-power view of lesion ② demonstrating a localized proliferation of cytologically bland thymic epithelial cells measuring less than 1 mm, consistent with microscopic thymoma. H&E, Hematoxylin and eosin.

The distinction between the two lesions was based on their overall histomorphological characteristics in addition to their size and was not made solely on the basis of a size threshold. Although both lesions consisted of bland thymic epithelial cells, the smaller lesion lacked a discrete thymoma-like architecture and was interpreted as NEH, whereas the larger lesion demonstrated a more organized, compact epithelial proliferation resembling Type A thymoma and was therefore classified as Type A microthymoma.

Because both lesions demonstrated characteristic histomorphological features and the diagnosis could be established on routine histopathological examination, additional immunohistochemical studies were not considered necessary for routine diagnostic classification. However, no immunohistochemical or molecular analyses were performed, and this limitation is addressed in the Discussion.

The postoperative course was uneventful, and the patient was discharged on postoperative day 4. Because the microscopic Type A microthymoma was completely excised with the adjacent thymic tissue, showed no evidence of destructive invasion or residual disease, and was extremely small and histologically bland, additional completion thymectomy was not considered necessary. The patient was therefore managed with radiological surveillance rather than additional surgery or adjuvant therapy. Follow-up chest CT was performed every six months after surgery. At the latest follow-up, 18 months after surgery, no radiological recurrence or newly developed mediastinal lesion was identified. The patient remains under clinical and radiological surveillance.

## Discussion

Thymic epithelial lesions may range from benign microscopic proliferations to clinically apparent thymic epithelial tumors. In this context, the distinction between nodular epithelial hyperplasia (NEH), historically referred to as “microscopic thymoma,” and microthymoma is particularly important. NEH represents a microscopic proliferation of thymic epithelial cells without sufficient architectural and cytological features to establish a diagnosis of thymoma, whereas microthymoma is a small thymic epithelial neoplasm that demonstrates histological features resembling those of conventional thymoma. Importantly, the distinction between these lesions should not be based on size alone but should also take into account their architectural and cytological characteristics.

Microthymoma was proposed as a distinct pathological entity by Cheuk et al., who described small nodular thymic epithelial proliferations that were distinct from nodular epithelial hyperplasia [1]. Type A microthymoma is characterized by a compact proliferation of bland spindle-shaped or oval thymic epithelial cells resembling those of conventional Type A thymoma. In contrast, lesions historically termed “microscopic thymoma” have generally been described as small, localized proliferations of thymic epithelial cells, often measuring less than 1 mm, but their biological significance remains uncertain [2]. In the fifth edition of the World Health Organization (WHO) Classification of Thoracic Tumours, these lesions are considered within the spectrum of microscopic thymic epithelial proliferations rather than conventional thymoma [3]. Previous studies have reported microscopic thymic epithelial proliferations predominantly in patients with myasthenia gravis, whereas microthymoma has also been identified in thymectomy specimens from patients with myasthenia gravis [4,5].

The coexistence of microscopic thymic epithelial proliferations and thymic cysts is uncommon. Furuya et al. reported a thymic cyst associated with a 1.6-mm Type A microthymoma in the surrounding thymic tissue, together with multiple hyperplastic nodules measuring less than 1 mm that were histologically interpreted as microscopic thymomas [6]. Thus, the association between a thymic cyst and both a Type A microthymoma and microscopic thymic epithelial proliferations has been previously documented (Table 2). Other reports have described microscopic thymic epithelial proliferations associated with thymic cysts, although the number of reported cases remains limited [7-9].

**Table 2:** Reported cases of microscopic thymoma associated with thymic cysts in patients without myasthenia gravis Published cases of microscopic thymoma associated with thymic cysts in patients without myasthenia gravis are summarized together with the present case. Findings of concomitant microthymoma are also included when available. In the present case, a 0.6-mm microscopic thymoma and a 2.0-mm Type A microthymoma were identified simultaneously within thymic tissue adjacent to the thymic cyst. M, male; F, female; MG, myasthenia gravis; N.A., not available.

Year	Author	Age/Gender	Cyst size (mm)	Tumor size	Diagnosis	Location vs cyst	MG	Treatment	Outcome
2018	Furuya et al.[6]	75/M	33	1.6mm <1mm	Micro & Microscopic	not reported	Absent	Partial resection of the thymus	1-year no recurrence
2024	Ono and Hara [7]	80/M	155	500μm	Microscopic	Within cyst wall	Absent	Partial resection of the thymus	N.A
2024	Komatsu et al. [8]	81/F	13	400μm	Microscopic	not reported	Absent	Resection	N.A
2026	PresenP case	54/F	40	2.0mm, 0.6mm	Micro & Microscopic	not reported	Absent	Partial resection of the thymus	1-year no recurrence

In the present case, the two microscopic lesions were located in the thymic tissue adjacent to the cyst wall, rather than within the cyst wall itself. This finding is consistent with the anatomical distribution described in several previous reports and suggests that careful examination of the residual thymic tissue surrounding a thymic cyst may be important for detecting these otherwise occult lesions.

The biological relationship between thymic cysts and microscopic thymic epithelial proliferations remains uncertain. Cystic changes are well recognized in conventional thymomas, and thymic cysts may occur in association with thymic epithelial tumors [10]. One possible explanation is that chronic inflammation, tissue remodeling, or epithelial injury associated with a thymic cyst may promote localized proliferation of thymic epithelial cells. Alternatively, microscopic epithelial proliferation may precede the development of cystic change. However, these possibilities remain speculative, and the present case does not establish a causal relationship between the cyst and the adjacent epithelial lesions.

The most notable pathological finding in the present case was the simultaneous identification of two morphologically distinct microscopic epithelial lesions, measuring 0.6 × 0.4 mm and 2.0 × 0.6 mm, respectively, within the same thymic tissue adjacent to the cyst. The smaller lesion was interpreted as NEH, historically termed “microscopic thymoma,” whereas the larger lesion demonstrated a compact, thymoma-like proliferation of bland spindle-shaped to oval epithelial cells and was diagnosed as Type A microthymoma. Although both lesions shared bland cytological features, their architectural patterns differed sufficiently to support separate pathological classifications. Thus, the present case illustrates that the distinction between NEH and microthymoma should be based on overall histomorphological features rather than solely on a size threshold.

The close anatomical proximity and overlapping cytological features of these two lesions raise the possibility that NEH and microthymoma may represent different points within a morphological spectrum of thymic epithelial proliferation. A hypothetical sequence in which a localized epithelial proliferation progresses from hyperplasia to a more organized thymoma-like proliferation is biologically conceivable. However, there is currently insufficient evidence to establish such a progression, and the present case cannot demonstrate a precursor-product relationship. The possibility that these lesions simply represent independent microscopic epithelial proliferations occurring within the same thymic environment must therefore also be considered. Additional cases with detailed immunohistochemical and molecular analyses will be necessary to determine whether these lesions share a common biological origin.

Immunohistochemical and molecular analyses were not performed in the present case because both lesions showed characteristic histomorphological features that allowed a confident diagnosis on routine histopathological examination. Nevertheless, this represents an important limitation of the present report. In particular, the absence of immunohistochemical and molecular data prevents further evaluation of the biological relationship between NEH and Type A microthymoma. Future studies incorporating markers of thymic epithelial differentiation and molecular profiling may help clarify whether these lesions represent biologically related or independent processes.

From a clinical perspective, there is currently insufficient evidence that either NEH or microthymoma identified incidentally in the setting of a thymic cyst independently affects prognosis or requires modification of the standard management of an otherwise benign thymic cyst. In the present case, complete resection of the cyst with the adjacent thymic tissue was performed, and no recurrence was observed during 18 months of follow-up. Although longer follow-up is required, the clinical course was favorable. Importantly, our findings do not support additional surgery solely because of the incidental identification of these microscopic lesions.

The present case therefore provides an additional example of the coexistence of a thymic cyst, NEH, and Type A microthymoma. Although a similar association has previously been reported, the simultaneous identification of two morphologically distinct microscopic thymic epithelial proliferations in close proximity within the same specimen further illustrates the diagnostic continuum and unresolved biological relationship between these lesions. Careful histopathological examination of residual thymic tissue surrounding resected thymic cysts may facilitate recognition of occult microscopic epithelial proliferations and improve pathological characterization of these uncommon lesions. Further accumulation of cases, together with immunohistochemical and molecular investigations, is warranted to clarify their biological significance and relationship.

## Conclusions

We report a rare case of nodular epithelial hyperplasia (NEH), historically termed “microscopic thymoma,” and Type A microthymoma occurring simultaneously in thymic tissue adjacent to a thymic cyst. Although these lesions shared bland cytological features and were located in close proximity, their distinct architectural characteristics supported separate pathological classification. This case underscores the importance of distinguishing NEH from microthymoma based on their overall histomorphological features rather than size alone. While the biological relationship between these microscopic thymic epithelial proliferations remains uncertain, careful histopathological examination of thymic tissue adjacent to resected thymic cysts may facilitate the detection and accurate characterization of otherwise occult lesions. Further accumulation of cases with immunohistochemical and molecular analyses will be necessary to clarify their biological significance and potential relationship.
